# A human-in-the-loop based Bayesian network approach to improve imbalanced radiation outcomes prediction for hepatocellular cancer patients with stereotactic body radiotherapy

**DOI:** 10.3389/fonc.2022.1061024

**Published:** 2022-12-09

**Authors:** Yi Luo, Kyle C. Cuneo, Theodore S. Lawrence, Martha M. Matuszak, Laura A. Dawson, Dipesh Niraula, Randall K. Ten Haken, Issam El Naqa

**Affiliations:** ^1^ Department of Machine Learning, Moffitt Cancer Center, Tampa, FL, United States; ^2^ Department of Radiation Oncology, University of Michigan, Ann Arbor, MI, United States; ^3^ Department of Radiation Oncology, University of Toronto, Toronto, ON, Canada

**Keywords:** accuracy and explainability, Bayesian networks, human-in-the-loop, hepatocellular cancer, outcome prediction, stereotactic body radiotherapy

## Abstract

**Background:**

Imbalanced outcome is one of common characteristics of oncology datasets. Current machine learning approaches have limitation in learning from such datasets. Here, we propose to resolve this problem by utilizing a human-in-the-loop (HITL) approach, which we hypothesize will also lead to more accurate and explainable outcome prediction models.

**Methods:**

A total of 119 HCC patients with 163 tumors were used in the study. 81 patients with 104 tumors from the University of Michigan Hospital treated with SBRT were considered as a discovery dataset for radiation outcomes model building. The external testing dataset included 59 tumors from 38 patients with SBRT from Princess Margaret Hospital. In the discovery dataset, 100 tumors from 77 patients had local control (LC) (96% of 104 tumors) and 23 patients had at least one grade increment of ALBI (I-ALBI) during six-month follow up (28% of 81 patients). Each patient had a total of 110 features, where 15 or 20 features were identified by physicians as expert knowledge features (EKFs) for LC or I-ALBI prediction. We proposed a HITL based Bayesian network (HITL-BN) approach to enhance the capability of selecting important features from imbalanced data in terms of accuracy and explainability through humans’ participation by integrating feature importance ranking and Markov blanket algorithms. A pure data-driven Bayesian network (PD-BN) method was applied to the same discovery dataset of HCC patients as a benchmark.

**Results:**

In the training and testing phases, the areas under receiver operating characteristic curves of the HITL-BN models for LC or I-ALBI prediction during SBRT are 0.85 (95% confidence interval: 0.75-0.95) or 0.89 (0.81-0.95) and 0.77 or 0.78, respectively. They significantly outperformed the during-treatment PD-BN model in predicting LC or I-ALBI based on the discovery cross-validation and testing datasets from the Delong tests.

**Conclusion:**

By allowing the human expert to be part of the model building process, the HITL-BN approach yielded significantly improved accuracy as well as better explainability when dealing with imbalanced outcomes in the prediction of post-SBRT treatment response of HCC patients when compared to the PD-BN method.

## 1 Introduction

Hepatocellular cancer (HCC) is the third leading cause of cancer death worldwide. In 2020, the American Society of Clinical Oncology (ASCO) estimated that 830,180 people around the world died from the disease. While radiotherapy is designed to achieve tumor local control (LC) in HCC patients, it may also lead to radiation-induced toxicities (RITs). As a relatively newer radiation treatment technique, stereotactic body radiation therapy (SBRT) uses focused beams of radiation aimed at the tumor from many different angles given in one to five treatment fractions. Thus, the aim of SBRT is to cure tumors in the meanwhile decreasing the radiation to nearby healthy tissues. While it is more effective for tumor LC and RITs reduction compared to conventional approaches, stringent dose volume constraints of SBRT require the treatment planning to be highly personalized to meet its intended goals (1).

In HCC SBRT, LC can be evaluated radiologically from a lesion that is no longer arterially enhancing and has not spread to neighboring lymph nodes without any failures within the irradiated area over long-term follow-up (2). The impact of RITs to baseline liver function of HCC patients before and after SBRT can be evaluated by albumin-bilirubin (ALBI) grades for personalized standard or adaptive implementation (3–5). Specifically, physicians are concerned whether patients’ ALBI grades will increase at least by one grade or not during 6-month follow-up, which is denoted as I-ALBI. Thus, we considered I-ALBI as another relevant SBRT outcome in addition to tumor LC in this study. The literature on outcomes prediction models for HCC patients with SBRT and their explainability capability remains limited and challenging (6, 7). The purpose of this study is to fill these gaps by developing accurate and explainable LC or I-ALBI prediction models for HCC patients with SBRT.

In clinical practice, oncology datasets usually have high dimensional features with limited sample size making susceptible to spurious correlations including the Simpson paradox (8). The dataset of HCC patients with SBRT in this study is not an exception. Machine learning (ML) is defined as the task of extracting information from possibly high-dimensional and noisy data to give some guarantees of performance on unseen data. However, extracting the structure based on the proximity between empirical and population densities becomes challenging in the higher dimensions, since the distance between objects may be heavily dominated by noise, and the associated optimization process has an exponential dependency on these dimensions (9). Then, feature selection is designed to help conventional ML approaches handle high-dimensional datasets. For example, in our previous study on personalized adaptive radiotherapy for non-small-cell lung cancer patients, a pure data-driven Bayesian network (PD-BN) approach is developed including feature selection and BN structure building two steps. While Markov blanket (MB) algorithms were employed in the first step to identify the most important features from high-dimensional oncology datasets, Tabu Search was used in the second step to learn network structure based on the selected features. In addition to unraveling the biophysical relationships among lung cancer patients’ personal characteristics, radiation treatment, and outcomes, the PD-BNs can predict lung tumor LC or/and RITs and identify the best treatment strategies before and during the radiotherapy to improve patients’ therapeutic satisfaction (10–12).

**Figure 1:**
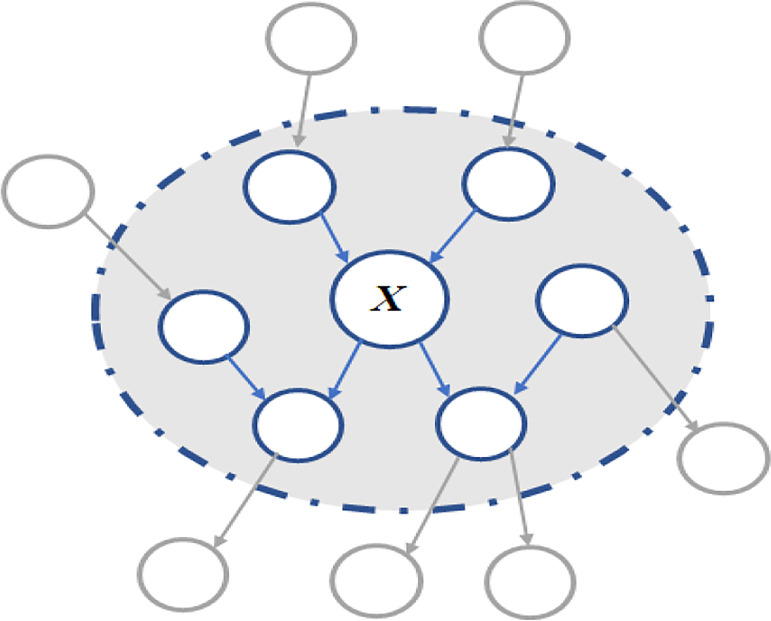
Markov blanket of variable *X*.

Initially proposed by Pearl (13), the concept of variable *X*’s MB is to identify its optimal feature subset containing strongly relevant and non-redundant features, such as the variable’s parents, children, and spouses as shown in the shadow area of **Figure 1**. Given these features in the subset, the variable is independent to other features outside it. Due to its capability of fully explaining a target variable, the MB has the potential of selecting the features that have strong relevance to an outcome for building its prediction models. Then the MB algorithms such as incremental association MB (14) and its variants (15) were successfully employed in the feature selection process of our previous PD-BN approach to develop accurate and interpretable outcome prediction models.

However, in addition to high dimensional features with limited sample size, oncology datasets usually have imbalanced outcomes, such as HCC patients’ LC or I-ALBI in this study. The prediction of these treatment outcomes can be modeled as a binary classification problem under supervised machine learning. Class imbalance occurs when the minority group, such as non-LC or I-ALBI, contains significantly fewer events samples than the majority group, such as LC or non-I-ALBI. Learning these imbalanced outcomes from high-dimensional datasets can be very difficult (15, 16), and non-standard machine learning methods are often guaranteed to achieve desirable results (14). Moreover, features selected from the above theoretically sound MB algorithms to have a strong relevance with an outcome may not be able to build the BN-based outcome prediction model with high accuracy, since accuracy and explainability are two different criteria for feature selection (17). Then, the PD-BN based outcome prediction models have a limited prediction performance in this case. Furthermore, the developed PD-BNs are not necessarily following physicians’ common practice knowledge, and unconfirmed biophysical interactions explored from the PD-BN approach can barely gain physicians’ trust for application in routine clinical decision making. Therefore, the goal of this study is to develop a new ML approach in handling imbalanced oncology data to improve the accuracy and explainability of predicting HCC patients’ outcomes with SBRT.

Building accurate and explainable outcome prediction models from high-dimensional imbalanced data is a complex process that requires nontrivial understanding of complex ML algorithms (18). Humans are typically involved in unstructured manner at various points in the processes of the model development, model training, and testing of the underlying ML algorithm implementation. Human-in-the-loop ML (HITL-ML) approaches are proposed to rather define a new type of structured interactions between humans and machine learning algorithms. Being developed initially from reinforcement learning, preference learning, and active learning, the HITL-ML is a hybrid of data-driven and knowledge-driven approach that integrates *a priori* expert knowledge (EK) into ML frameworks to overcome issues related to model bias and uncertainty (19). In addition to making ML more accurate or to obtain the desired accuracy faster, the HITL-ML approach makes humans more effective and efficient (18). Especially, it is useful in handling imbalanced data (20, 21). Due to the transparency of the BN for potential clinical causal inferences, in this study we develop an HITL-BN approach to build HCC SBRT outcome prediction models from imbalanced oncology data by incorporating EK features and allowing human agents to participate in the BN feature selection process. The accuracy and explainability of HITL-BN based outcome prediction models are evaluated and compared to the PD-BN based models that do not involve human agents.

The rest of paper is organized as follows. Section 2 introduces the properties of our dataset and the details of the HITL-BN approach. Section 3 shows and compares outcome prediction models developed from the PD-BN and HITL-BN approaches. Section 4 discusses the accuracy and explainability of our new approach and verifies the relationships among biophysical features in developed HITL-BNs based on related literatures. Section 5 concludes our paper.

## 2 Material and methods

### 2.1 Participation and data collection

Our study uses 81 HCC patients with SBRT on prospective protocols under institutional review board (IRB) approval from University of Michigan Hospital (Michigan Medicine). Since each patient may have one or more tumors, there are totally 104 tumors in our discovery dataset. In this study, two or more tumors in an HCC patient are assumed to be independent from each other for the sake of simplicity. There are 23 patients with I-ALBI during six-month follow up, and 100 tumors from 77 patients achieved LC. Each patient has 97 features, including dosimetric information, clinical factors, pre- and during-treatment labs and cytokines as summarized in **Table 1**. The change of a lab or cytokine value during treatment was calculated from the difference between its post treatment (or three months after treatment) and pre-treatment (or baseline) values, and it is formulated by adding prefix “D_” to its name in our study. To avoid confusion in outcome prediction, biophysical features related to LC or I-ALBI were specified and manually designated by a human expert. For example, “gross tumor volume (GTV)” is considered for predicting LC instead of I-ALBI. The number of features for LC or I-ALBI prediction before and during SBRT are listed in **Table 2**. For independent external validation, we tested our developed models on a dataset from the Princess Margaret Hospital, which included 59 tumors from 38 HCC patients.

**Table 1:** Features of HCC patients with SBRT in the discovery dataset.

Categories	Names
Clinical Factors (25 features)	Sex, Age, pre-treatment_Cirrhosis (pre_Cirrhosis), Portal_Vein_Thrombosis, pre_SBRT, pre_SBRT_Liver, Active_Extrahepatic_Disease, Prior_Liver_Occurrences, Previously_Treated, Active_Liver_Lesions, Tumor_Size, gross tumor volume (GTV), planning target volume (PTV), Fiducials, Initial_Fraction, treatment break (Tx_Break) (22), Adapted, Total_Time, Break_Period, Number_of_Initial_Fractions (N_Initial_Fractions), N_Final_Fractions, Total_N_Fractions, Liver_GTV_Volume, pre-treatment eastern cooperative oncology group performance status (pre_ECOG_PS), D_ECOG_PS
Dosimetric Information (35 features)	Initial_Dose, biologically effective dose (BED)_Initial_Dose, equivalent dose in 2 Gy fractions (EQD2)_Initial_Dose, Total_SBRT_Dose, Total_BED, Total_EQD2, BED_Manual (23), dose that covers 98% of GTV (GTV_D98), generalized equivalent uniform dose of GTV (GTV_gEUD), GTV_Min_Dose, dose that covers 98% of GTV using linear-quadratic (LQ) model (GTV_D98_LQ), GTV_gEUD_LQ, GTV_Mean_Dose_LQ, GTV_Min_Dose_LQ, dose that covers 98% of GTV using linear-quadratic-linear (LQL) model with threshold dose 20Gy (GTV_D98_LQL_DT20), gEUD of GTV with a= -20 using LQL model with α/β=10 (GTV_gEUD_N20_LQL_10**)**, GTV_Mean_Dose_LQL_DT20, GTV_Min_Dose_LQL_DT20, PTV_Mean_Dose, dose that covers 98% of PTV (PTV_D98), PTV_gEUD, PTV_Min_Dose, PTV_D98_LQ, PTV_gEUD_LQ, PTV_Mean_Dose_LQ, PTV_Min_Dose_LQ, PTV_D98_LQL_DT20, PTV_gEUD_LQL_DT20, PTV_Mean_Dose_LQL_DT20, PTV_Min_Dose_LQL_DT20, mean dose of total liver excluding gross tumor volume (LIVER_GTV_Mean_Dose), LIVER_GTV_Mean_Dose_LQ, LIVER_GTV_Mean_Dose_LQL, the 700 cm^3^ subvolume EQD2 of LIVER_GTV using LQ model with α/β = 2.5 Gy (LIVER_GTV_DC_LQ_EQD2) (24), the ≤ 15 Gy cold volume EQD2 of LIVER_GTV using LQ model with α/β = 2.5 Gy (LIVER_GTV_CV_EQD2_LQ) (24)
Pre- and During- Treatment Labs (27 features)	pre_Na, D_Na*, pre_Creatinine, D_Creatinine, pre_Albumin, D_Albumin, pre-treatment aspartate aminotransferase (pre_AST), D_AST, pre-treatment alanine transaminase (pre_ALT), D_ALT, pre_Alkphos, D_Alkphos, pre_Bilirubin, D_Bilirubin, pre-treatment international normalized ratio (pre_INR), D_INR, pre_Protime_INR, D_Protime_INR, pre-treatment alpha fetoprotein (pre_AFP), D_AFP, baseline model for end-stage liver disease (MELD_baseline), D_MELD, MELD_Na_baseline, D_MELD_Na, Child_Pugh, D_Child_Pugh, Barcelona_Score
Pre- and During- Treatment Cytokines (10 features)	pre-treatment indocyanine green after 15 minutes (pre_ICGR15) (25, 26), D_ICGR15, pre-treatment transforming growth factor beta (pre_TGF_Beta), D_TGF_Beta, pre-treatment CD40 ligand (pre_CD40_L) (27, 28), D_CD40_L, pre-treatment hepatocyte growth factor (pre_HGF) (28), D_HGF, pre_Eotaxin, D_Eotaxin

*”D_” is a prefix to indicate the change of during-treatment labs or cytokines, which was calculated from the difference of their values between post-treatment (or three months after treatment) and pre-treatment (or baseline).

**Table 2:** The number of features associated with each of 104 tumors before and during treatment for I-ALBI or LC prediction.

Outcome	I-ALBI		LC	
Time	Pre- Treatment	During Treatment	Pre- Treatment	During Treatment
**# of features associated with each tumor**	45	69	68	93

Physicians’ acquired knowledge and experience from treating HCC SBRT patients were collected and treated as expert knowledge (EK) for SBRT outcomes prediction in this study. The EK features (EKFs) related to I-ALBI prediction comprise “*LIVER_GTV_Mean_Dose*”, “*pre_ICGR15*”, “*D_ICGR15*”, “*Child_Pugh*”, “*Cirrhosis*”, “*Liver_GTV_Volume*”, “*pre_HGF*”, “*D_HGF*”, “*Age*”, “*Sex*”, “*pre_CD40_L*”, “*D_CD40_L*”. The EKFs for LC prediction include “*Child_Pugh*”, “*GTV*”, “*Total_BED*”, “*LIVER_GTV_Mean_Dose*”, “*Prior_Liver_Occurrences*”, “*pre_MELD*”, “*pre_Bilirubin*”, “*pre_Albumin*”, “*Tumor_Size*”, “*GTV_Mean_Dose_LQ*”, “*pre_ICGR15*”, “*pre_HGF*”, and “*pre_TGF_Beta*”. Except the above EKFs related to two different outcomes, the rest of features in **Table 1** were denoted as non-EK features (non-EKFs) in this study.

### 2.2 Human-in-the-loop to handle imbalanced data in feature selection

Gained from many years of experience, reading articles, training, peers’ interaction, EK has a potential of bypassing otherwise complex systems and providing parsimonious solutions that focus on key aspects of a given situation. By incorporating EKFs into the feature selection of the PD-BN approach, we previously developed a situational awareness BN (SA-BN) method to predict the radiation outcomes of lung cancer patients (29). With the SA-BNs, the physicians not only are able to know patients’ situation and predict LC and potential RITs starting from their acquired knowledge, but also can evaluate the best treatment strategies to maximize the LC and minimize the RITs before and during the course of radiotherapy. Focusing on improving the PD-BN based models’ explainability, the SA-BN method has limitations in alleviating the impact of high-dimensional imbalanced data on the PD-BN approach’s feature selection process to improve the accuracy of its associated outcome prediction models (29). However, the tighter confidence intervals of prediction performance and well-known biophysical relationships in the SA-BNs indicated that the EK has potential to improve the accuracy and explainability of outcome prediction models. Then, the EK methodology was employed in the HITL-BN approach to guide feature selection from imbalanced HCC SBRT data.

Selecting an ML approach for outcome prediction often involves a trade-off between prediction accuracy and explainability (30). While some ML approaches may lead to relatively more accurate outcome prediction models, other ML methods can result in more explainable ones. According to the explainability of their associated outcome prediction models, the ML approaches can be generally classified into explainable ML (EML) and unexplainable ML (UML) methods. The former includes Decision Trees, Logistic Regression and its variants, Naïve BNs, BNs, etc., and the latter comprises Random Forests (RFs), Support Vector Machines (SVMs), Gradient Boosting Machines (GBMs), Deep Learning (DL), etc. Although the EML-based outcome prediction models generally have relatively lower prediction accuracy compared to the UML-based models, they can be used to identify the most relevant features in explaining an outcome. On the other hand, while the UML-based outcome prediction models have difficulties in interpreting the relationships between specific features and the outcome, a list of ranked features can be generated from each of them based on features’ importance in terms of outcome prediction (31). However, the ranking lists generated from different UML approaches may not be the same, resulting in different important features selected from the top rank of these lists for outcome prediction. An integrated feature ranking list is developed in this study by combining these lists based on the performance of its associated UML-based outcome prediction models to achieve robust feature selection as introduced in the next section.

The selected features from the EML and UML approaches are generally different, even though they are evaluated from one single dataset. The former and latter have the potential of improving an outcome prediction model’s explainability and accuracy respectively. While the MB algorithm and network structure learning were considered as a *computational agent* to improve the prediction model’s explainability by exploring EKFs and non-EKFs that have strong relevance to an outcome, the integrated feature ranking list was treated as another *computational agent* to enhance the prediction model’s accuracy by investigating each feature’s importance in terms of outcome prediction. Then, the HITL-BN approach can improve its capability of learning from the imbalanced HCC SBRT data by allowing human agents to interact these two computational agents during the process of feature selection.

### 2.3 The human-in-the-loop BN approach

As stated previously, the UML approaches include RF, SVM, GBM, DL, etc., and each of them can generate a feature ranking list in terms of importance in outcome prediction from all features including EKFs and non-EKFs in a dataset. Let *K* be the total kinds of these UML approaches, *k* be the index of these approaches (*k* =1, 2, 3, …, *K*), *L* ^*k*^ be a feature ranking list obtained from UML approach *k* (*k* =1, 2, 3, …, *K*) with the most important feature for outcome prediction at the top of the list, and *AUC* ^*k*^ be the performance of an outcome prediction model developed from UML approach *k* based on cross validation in the discovery dataset (*k* =1, 2, 3, …, *K*). Let *J* be the total number of features in the discovery dataset, *j* be the index randomly assigned to them (*j* = 1, 2, 3, …, *J*), *N*_*j*_(*L* ^*k*^) be the rank of feature *j* in ranking list *L* ^*k*^ (*j* = 1, 2, 3, …, *J*, *k* =1, 2, 3, …, *K*). The rankings *N*_*j*_(*L* ^*k*^) of the feature in different lists *L* ^*k*^ may not be the same, and the performance *AUC* ^*k*^ of UML approaches for outcome prediction could be different. It is assumed that a robust feature ranking list can be developed by integrating all these ranking lists based on their corresponding UML approaches’ prediction performance. Let *L* ^*^ be an integrated feature ranking list based on *K* UML approaches, be the weighted ranking score (WRS) of feature *j* to determine its ranking in list *L* ^*^ , and its value can be evaluated from the following equation by integrating its ranks *N*_*j*_(*L* ^*k*^) in different ranking lists *L* ^*k*^



*j* = 1, 2, 3, …, *J* (1) $$WRS_{j}=\sum_{k=1}^{K}\frac{N_{j}\left(L^{k}\right)*\sum_{k=1}^{K}AUC^{k}}{AUC^{k}}$$

Then, the ranking list *L* ^*^ in terms of features’ importance in outcome prediction can be obtained from sorting all the features based on their *WRS*_*j*_ , where the feature with the minimal score value is ranked at the top of the list.

Including feature selection and BN structure learning processes, HITL-BN based outcome prediction models are mainly developed based on the integrated ranking list. Let *I* be the total number of EKFs in list *L* ^*^ with *I*<*J* , *i* represent the order of an EKF within all EKFs (*i* = 1, 2, 3, …, *I*). An initial HITL-BN is developed from the top *n* percent of features in the list. The value of *n* depends on the total number *N* of features in a dataset and appropriate feature dimension *D* to satisfy the MB algorithms’ faithfulness assumption, and we assumed *n* = 100* DN . Suppose the top *n* percent of features in list includes *i* EKFs (*i*≤*D* ), an initial HITL-BN based outcome prediction model can be denoted as HITL-BN(*i*). Since some EKFs in the top rank of list *L* ^*^ may be redundant or less relevance to an outcome compared to other ones, the most relevant EKFs can be identified from the outcome’s MB. Given the selected EKFs, important non-EKFs in the top rank of the list to improve the outcome prediction should also be strongly related to these EKFs, which can be identified from each of their MBs. Thus, important EKFs and non-EKFs can be selected from the top rank of the list with balanced accuracy and explainability for HITL-BN(*i*) development. Note that the structure learning of HITL-BN(*i*) is the same as that of PD-BNs, where less important EKFs and non-EKFs are eliminated from the network to maximize its prediction performance. The rest of our HITL-BN approach is to repeatedly evaluate whether a next EKF and additional non-EKFs before it in list *L* ^*^ can improve the accuracy of previous outcome prediction models or not.

**Figure 2:**
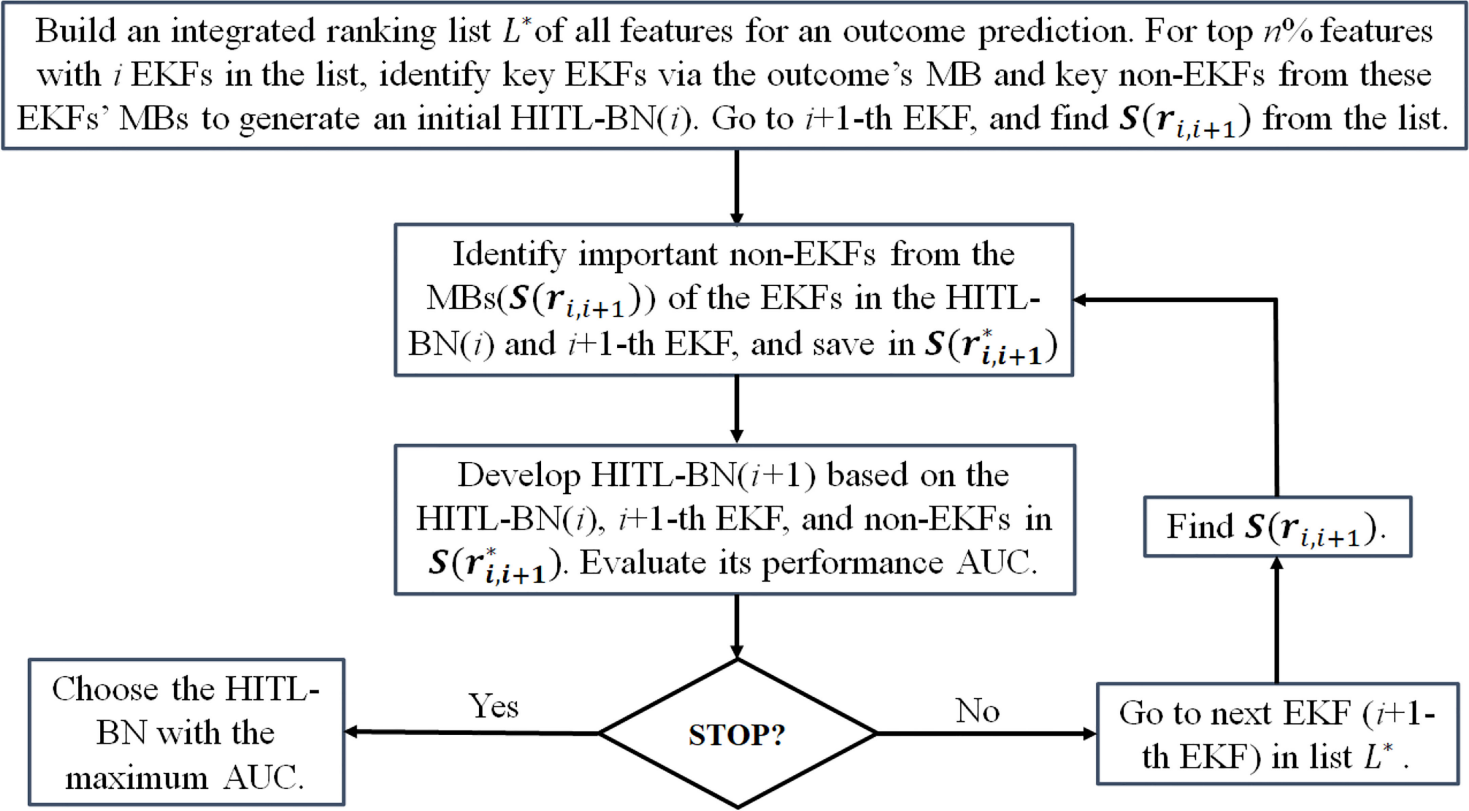
The flow chart of the HITL-BN approach.

Let *r*_*i*_ be the rank of *i-*th EKF in list *L* ^*^ (*i* =1, 2, 3, …, *I*). As the evaluation moves from *i-*th EKF to *i*+1-th EKF in the integrated feature ranking list, the set of additional indices between them is denoted as *r*_*i*, *i*+1_ . Let *S*(*r*_*i*, *i*+1_) represents the set of non-EKFs associated with *r*_*i*, *i*+1_ , and the number of non-EKFs in the set could be zero when two EKFs are consecutive in the list. If set *S*(*r*_*i*, *i*+1_) is not empty, the importance of these non-EKFs for the outcome prediction depends on whether they have strong relevance with selected EKFs, including the EKFs in HITL-BN(*i*) and *i*+1-th EKF. Let MBs (*S*(*r*_*i*,*i*+1_) ) be these EKFs’ MBs based on non-EKFs in *S*(*r*_*i*,*i*+1_) , and the set of selected non-EKFs from these MBs is indicated as $S\left(r_{i,i+1}^{*}\right)$ . Then HITL-BN(*i*+1) can be developed based on *i*+1-th EKF, non-EKFs in $S\left(r_{i,i+1}^{*}\right)$ together with all the features in HITL-BN(*i*) by employing PD-BN’s structure learning process. The process continues along list until the performance of prediction model cannot be improved or meet a target prediction performance. The details of the HITL-BN approach to generate an accurate and explainable outcome prediction model are described in **Figure 2**.

## 3 Results

### 3.1 PD-BN models for I-ALBI or LC prediction

**Figure 3:**
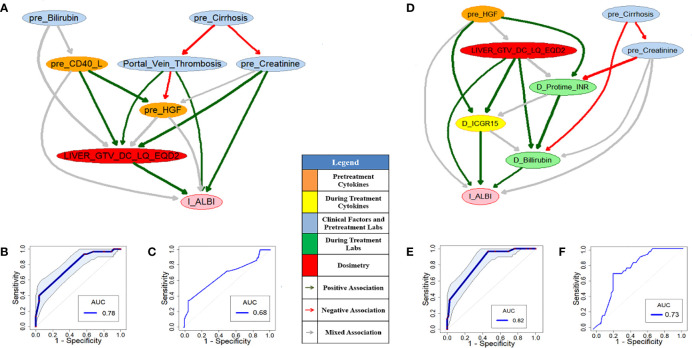
Pre- **(A)** and during-treatment **(D)** PD-BNs for I-ALBI prediction. The prediction performance of pre- **(B)** and during-treatment **(E)** PD-BNs based on the discovery dataset. The prediction performance of pre- **(C)** and during-treatment **(F)** PD-BNs based on the testing dataset.

**Figure 4:**
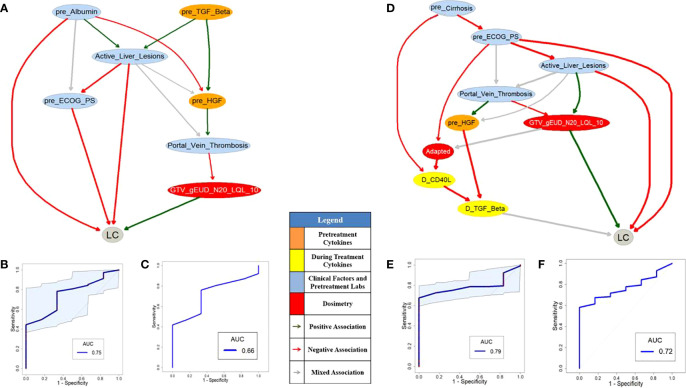
Pre- **(A)** and during-treatment **(D)** PD-BNs for LC prediction. The prediction performance of pre- **(B)** and during-treatment **(E)** PD-BNs based on the discovery dataset. The prediction performance of pre- **(C)** and during-treatment **(F)** PD-BNs based on the testing dataset.

As a comparison of the HITL-BN approach, PD-BN models for I-ALBI or LC prediction were developed based on our HCC SBRT patients as shown in **Figures 3** or **4**. Numerical experiments in this study were conducted in an R environment, where function “inter-MB” in R package “bnlearn” was employed as the MB algorithm for feature selection and function “boot.strength” in the same R package was used for BN structure learning. **Figures 3A** or **3D** shows pre- or during-treatment PD-BN model for I-ALBI prediction developed from the discovery dataset. While the PD-BN method selected biophysical features “*pre_Bilirubin*”, “*pre_Cirrhosis*”, “*Portal_Vein_Thrombosis*”, “*pre_Creatinie*”, “*pre_CD40_L*”, “*pre_HGF*”, and “*Liver_GTV_DC_LQ_EQD2*” for pre-treatment I-ALBI prediction, additional variables “*D_Protime_INR*”, “*D_Bilirubin*”, and “*D_ICGR15*” were chosen for during-treatment I-ALBI prediction. The prediction performances of the former and the latter based on the discovery dataset are 0.78 (95%CI: 0.67-0.83) and 0.82 (95%CI: 0.74-0.88) as described in **Figures 3B** and **3E** respectively. The prediction performance of the former or the latter based on the testing dataset is 0.68 or 0.73 as illustrated by **Figures 3C** or **3F**.

**Figures 4A** or **4D** shows pre- or during-treatment PD-BN for LC prediction generated from the discovery dataset. While the PD-BN method selected features “*pre_Albumin*”, “*Active_Liver_Lesions*”, “*Portal_Vein_Thrombosis*”, “*pre_ECOG_PS*”, “*pre_TGF_Beta*”, “*pre_HGF*”, and “*GTV_gEUD_N20_LQL_10*” for pre-treatment LC prediction, additional variables “*pre_Cirrhosis*”, “*Adapted*”, “*D_CD40_L*”, and “*D_TGF_Beta*” were chosen for during-treatment LC prediction. The prediction performances of the former and the latter based on the discovery dataset are 0.75 (95%CI: 0.60-0.86) and 0.79 (95%CI: 0.69-0.89) as shown in **Figures 4B** and **4E** respectively. The prediction performance of the former or the latter based on the testing dataset is 0.66 or 0.72 as illustrated by **Figures 4C** and **4F**.

### 3.2 HITL-BN models for HCC SBRT patients’ outcomes prediction

We conducted numerical experiments to develop or test HITL-BN models for I-ALBI or LC prediction based on the discovery and testing datasets in the same R environment as that of developing or testing the PD-BN models. Two UML approaches, the RF and GBM (*K*=2), were employed in this study to generate an integrated feature ranking list for a HITL-BN based outcome prediction model development before or during treatment. RF and GBM share similar tree/graph structure learning to BN. Packages ‘randomForestSRC’ and ‘gbm’ were used to identify feature ranking lists from the former and latter approaches based on the discovery dataset respectively. After evaluating the two UML approaches’ prediction performances, each feature’s WRS was computed based on its ranks in two different ranking lists and the corresponding UML approaches’ prediction performances from Equation (1). Then, an integrated feature ranking list to rank all the features in the discovery dataset for I-ALBI or LC prediction before or during SBRT can be generated from their WRSs.

#### 3.2.1 HCC SBRT patients’ I-ALBI prediction

**Figure 5:**
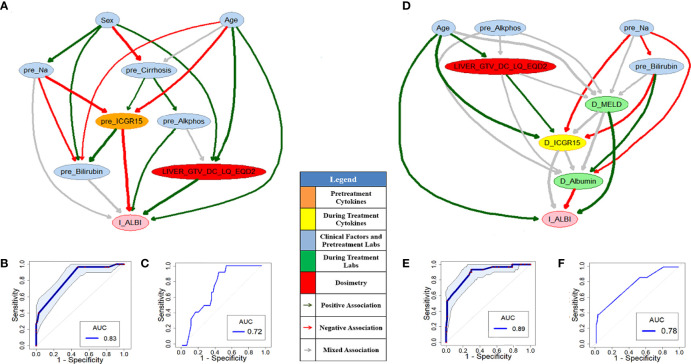
Pre- **(A)** and during-treatment **(D)** HITL-BNs for I-ALBI prediction. The prediction performance of pre- **(B)** and during-treatment **(E)** HITL-BNs based on the discovery dataset. The prediction performance of pre- **(C)** and during-treatment **(F)** HITL-BNs based on the testing dataset.

**Figures 5A** or **5D** shows pre- or during-treatment HITL-BN for I-ALBI prediction developed from the discovery dataset. While the HITL-BN approach selected features “*Sex*”, “*Age*”, “*pre_Na*”, “*pre_Cirrhosis*”, “*pre_Alkphos*”, “*pre_Billirubin*”, “*pre_ICGR15*”, and “*LIVER_GTV_DC_LQ_EQD2*” for pre-treatment I-ALBI prediction, additional variables “*D_MELD*”, “*D_Albumin*”, and “*D_ICGR15*” were chosen for during-treatment I-ALBI prediction. **Tables 3** and **4** show the integrated feature ranking lists of all features according to their WRSs for I-ALBI prediction before and during SBRT respectively. The features in PD-BNs as shown in **Figure 3** are highlighted with *italic* font in these tables, and the features in HITL-BNs as illustrated in **Figure 5** are emphasized with **bold** font in them. Especially, the features marked with italic and bold fonts come from both the PD-BN and HITL-BN.

**Table 3:** The rank of features in an integrated feature ranking list for pre-treatment I-ALBI prediction.

Rank	Feature Names	Rank	Feature Names	Rank	Feature Names
1	pre_Albumin	16	Total_SBRT_Dose	31	Prior_Liver_Occurences
2	**pre_ICGR15**	17	Total_EQD2	32	EQD2_Initial_Dose
3	**pre_Alkphos**	18	LIVER_GTV_Mean_Dose_LQ	33	MELD_baseline
4	**pre_Na**	19	BED_Manual	34	Initial_Dose
5	**Age**	20	pre_AST	35	**Sex**
6	***LIVER_GTV_DC_LQ_EQD2***	21	pre_Eotaxin	36	Active_Liver_Lesions
7	LIVER_GTV_CV_EQD2_LQ	22	Liver_GTV_Volumn	37	Barcelona_Score
8	***pre_Bilirubin***	23	pre_Protime_INR	38	Child_Pugh
9	pre_AFP	24	LIVER_GTV_Mean_Dose	39	Fiducials
10	*pre_Creatinine*	25	BED_Initial_Dose	40	pre_INR
11	Total_BED	26	LIVER_GTV_Mean_Dose_LQL	41	pre_ECOG_PS
12	*pre_CD40_L*	27	***pre_Cirrhosis***	42	Active_Extrahepatic_Disease
13	pre_TGF_Beta	28	Initial_Fraction	43	pre_SBRT
14	*pre_HGF*	29	*Portal_Vein_Thrombosis*	44	Previously_Treated
15	MELD_Na_baseline	30	pre_ALT	45	pre_SBRT_Liver

**Table 4:** The rank of features in an integrated feature ranking list for during-treatment I-ALBI prediction.

Rank	Feature Name	Rank	Feature Name	Rank	Feature Name
1	**pre_Alkphos**	24	*pre_Creatinine*	47	pre_ALT
2	pre_ICGR15	25	*pre_HGF*	48	Prior_Liver_Occurences
3	pre_Albumin	26	pre_TGF_Beta	49	Break_Period
4	**D_MELD**	27	pre_AST	50	LIVER_GTV_Mean_Dose_LQL
5	***D_ICGR15***	28	D_INR	51	Child_Pugh
6	**pre_Na**	29	D_ALT	52	Barcelona_Score
7	D_Creatinine	30	MELD_Na_baseline	53	Portal_Vein_Thrombosis
8	**pre_Bilirubin**	31	D_TGF_Beta	54	Total_Time
9	D_HGF	32	*D_Bilirubin*	55	Total_EQD2
10	D_Eotaxin	33	MELD_baseline	56	D_ECOG_PS
11	***LIVER_GTV_DC_LQ_EQD2***	34	Total_SBRT_Dose	57	Total_N_Fractions
12	pre_Protime_INR	35	Liver_GTV_Volumn	58	D_Child_Pugh
13	D_Alkphos	36	pre_Eotaxin	59	N_Final_Fractions
14	D_CD40_L	37	D_Na	60	Tx_Break
15	D_AFP	38	Initial_Fraction	61	Sex
16	D_MELD_Na	39	Total_BED	62	Active_Extrahepatic_Disease
17	pre_AFP	40	Initial_Dose	63	Fiducials
18	**D_Albumin**	41	BED_Manual	64	pre_SBRT
19	*D_Protime_INR*	42	LIVER_GTV_Mean_Dose_LQ	65	pre_ECOG_PS
20	**Age**	43	BED_Initial_Dose	66	Active_Liver_Lesions
21	LIVER_GTV_CV_EQD2_LQ	44	LIVER_GTV_Mean_Dose	67	pre_INR
22	pre_CD40_L	45	EQD2_Initial_Dose	68	Previously_Treated
23	D_AST	46	*pre_Cirrhosis*	69	pre_SBRT_Liver

The performances AUCs of pre- and during-treatment HITL-BNs for I-ALBI prediction based on the discovery dataset are 0.83 (95%CI: 0.75-0.89) and 0.89 (95%CI: 0.81-0.95) as shown in **Figures 5B**, **E** respectively. While the performance of the former is not significantly better than that of pre-treatment PD-BN as illustrated in **Figure 3A**, the latter significantly outperforms during-treatment PD-BN as shown in **Figure 3D** based on the DeLong test with *p*-value=0.0253. For the testing dataset, the performance of pre- or during-treatment HITL-BN for I-ALBI prediction is 0.72 or 0.78 as illustrated by **Figures 5C** or **5F**, and the latter significantly outperforms during-treatment PD-BN from the Delong test with *p*-value=0.0318.

#### 3.2.2 HCC SBRT patients’ LC prediction

**Figure 6:**
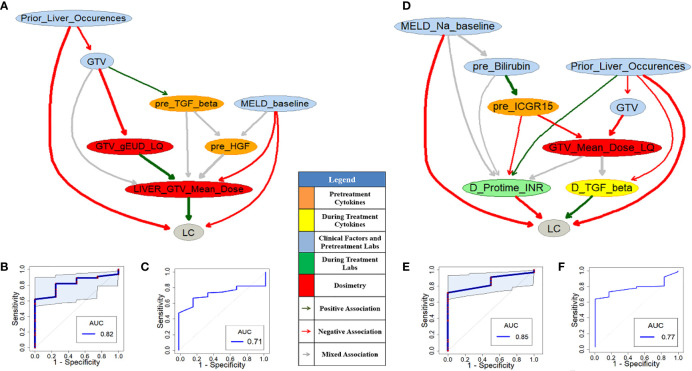
Pre- **(A)** and during-treatment **(D)** HITL-BNs for LC prediction. The prediction performance of pre- **(B)** and during-treatment **(E)** HITL-BNs based on the discovery dataset. The prediction performance of pre- **(C)** and during-treatment **(F)** HITL-BNs based on the testing dataset.

**Figures 6A** or **6D** shows pre- or during-treatment HITL-BN for LC prediction developed from the discovery dataset. While the HITL-BN for LC prediction approach selected features “*Prior_Liver_Occurences*”, “*GTV*”, “*MELD_baseline*”, “*pre_TGF_Beta*”, “*pre_HGF*”, “*GTV_gEUD_LQ*”, and “*LIVER_GTV_Mean_Dose*” for pre-treatment LC prediction, additional variables “*MELD_Na_baseline*”, “*pre_Billirubin*”, “*pre_ICGR15*”, “*GTV_Mean_Dose_LQ*”, “*D_Protime_INR*”, and “*D_TGF_Beta*” were chosen for during-treatment LC prediction. **Tables 5** and **6** show the ranking lists of all features according to their WRSs for LC prediction before and during SBRT respectively. The features from the PD-BNs as shown in **Figure 4** are highlighted with *italic* font in these tables, and the features from the HITL-BNs are emphasized with **bold** font in them. Especially, the features marked with italic and bold fonts come from both the PD-BN and HITL-BN.

**Table 5:** The rank of features in an integrated feature ranking list for pre-treatment LC prediction.

Rank	Feature Names	Rank	Feature Names	Rank	Feature Names
1	pre_AST	24	GTV_D98_LQ	47	GTV_Mean_Dose_LQ
2	PTV	25	pre_ICGR15	48	GTV_gEUD
3	pre_Protime_INR	26	PTV_D98	49	pre_SBRT
4	MELD_Na_baseline	27	GTV_Min_Dose_LQ	50	EQD2_Initial_Dose
5	***pre_TGF_Beta***	28	GTV_Mean_Dose	51	BED_Initial_Dose
6	PTV_gEUD_LQ	29	GTV_Min_Dose	52	Sex
7	**MELD_baseline**	30	pre_Eotaxin	53	Initial_Fraction
8	PTV_Min_Dose	31	**GTV_gEUD_LQ**	54	Age
9	**LIVER_GTV_Mean_Dose**	32	GTV_Mean_Dose_LQL_DT20	55	Barcelona_Score
10	**GTV**	33	*pre_Albumin*	56	pre_ALT
11	pre_INR	34	**Prior_Liver_Occurences**	57	*pre_ECOG_PS*
12	***pre_HGF***	35	PTV_D98_LQL_DT20	58	Fiducials
13	pre_AFP	36	*GTV_gEUD_N20_LQL_10*	59	Previously_Treated
14	PTV_Min_Dose_LQL_DT20	37	PTV_D98_LQ	60	Active_Extrahepatic_Disease
15	PTV_gEUD	38	PTV_Mean_Dose_LQL_DT20	61	GTV_D98
16	PTV_Min_Dose_LQ	39	pre_Na	62	*Portal_Vein_Thrombosis*
17	PTV_gEUD_LQL_DT20	40	pre_Bilirubin	63	pre_Cirrhosis
18	BED_Manual	41	Child_Pugh	64	Initial_Dose
19	pre_CD40_L	42	*Active_Liver_Lesions*	65	pre_SBRT_Liver
20	LIVER_GTV_CV_EQD2_LQ	43	PTV_Mean_Dose_LQ	66	Tumor_Size
21	Total_EQD2	44	PTV_Mean_Dose	67	pre_Alkphos
22	Total_BED	45	GTV_Min_Dose_LQL_DT20	68	pre_Creatinine
23	Total_SBRT_Dose	46	GTV_D98_LQL_DT20		

**Table 6:** The rank of features in an integrated feature ranking list for during-treatment LC prediction.

Rank	Feature Names	Rank	Feature Names	Rank	Feature Names
1	pre_AST	32	Total_EQD2	63	D_Albumin
2	PTV	33	GTV_Min_Dose_LQL_DT20	64	D_ECOG_PS
3	**MELD_Na_baseline**	34	PTV_D98_LQL_DT20	65	Initial_Dose
4	pre_Protime_INR	35	D_Bilirubin	66	Barcelona_Score
5	***D_TGF_Beta***	36	D_Eotaxin	67	Child_Pugh
6	LIVER_GTV_Mean_Dose	37	PTV_D98	68	D_ALT
7	PTV_Min_Dose	38	Total_BED	69	pre_Na
8	MELD_baseline	39	GTV_Min_Dose_LQ	70	BED_Initial_Dose
9	pre_TGF_Beta	40	D_AST	71	*pre_ECOG_PS*
10	**GTV**	41	GTV_Mean_Dose	72	Fiducials
11	D_HGF	42	D_Creatinine	73	N_Final_Fractions
12	PTV_gEUD	43	D_MELD	74	Total_N_Fractions
13	pre_AFP	44	GTV_Mean_Dose_LQL_DT20	75	N_Initial_Fractions
14	Total_Time	45	pre_Albumin	76	*Active_Liver_Lesions*
15	D_ICGR15	46	pre_Alkphos	77	**Prior_Liver_Occurences**
16	D_AFP	47	pre_ALT	78	Previously_Treated
17	PTV_Min_Dose_LQL_DT20	48	D_Na	79	Active_Extrahepatic_Disease
18	**D_Protime_INR**	49	BED_Manual	80	*Adapted*
19	PTV_gEUD_LQ	50	pre_Creatinine	81	Break_Period
20	D_MELD_Na	51	PTV_Mean_Dose_LQL_DT20	82	Age
21	*pre_HGF*	52	PTV_D98_LQ	83	pre_SBRT
22	pre_CD40_L	53	PTV_Mean_Dose	84	*Portal_Vein_Thrombosis*
23	pre_INR	54	*GTV_gEUD_N20_LQL_10*	85	*pre_Cirrhosis*
24	LIVER_GTV_CV_EQD2_LQ	55	GTV_D98_LQL_DT20	86	Sex
25	GTV_Min_Dose	56	**pre_Bilirubin**	87	*D_CD40_L*
26	PTV_Min_Dose_LQ	57	GTV_gEUD_LQ	88	pre_SBRT_Liver
27	**pre_ICGR15**	58	GTV_D98_LQ	89	Initial_Fraction
28	D_INR	59	GTV_gEUD	90	D_Child_Pugh
29	PTV_gEUD_LQL_DT20	60	GTV_D98	91	D_Alkphos
30	**GTV_Mean_Dose_LQ**	61	Tumor_Size	92	PTV_Mean_Dose_LQ
31	pre_Eotaxin	62	Tx_Break	93	

The performances of pre- and during-treatment HITL-BNs for LC prediction based on the discovery dataset are 0.82 (95%CI: 0.67-0.93) and 0.85 (95%CI: 0.75-0.95) as shown in **Figures 6B and 6E** and **6E** respectively. While the performance of the former is not significantly better than that of pre-treatment PD-BN as illustrated in **Figure 4A**, the latter significantly outperforms the during-treatment PD-BN as shown in **Figure 4D** based on the DeLong test with *p*-value=0.0367. For the testing dataset, the performance of pre- or during-treatment HITL-BN for LC prediction is 0.71 or 0.77 as illustrated by **Figure 6C** or **6F**, and the latter significantly outperforms the during-treatment PD-BN from the Delong test with *p*-value=0.0406. The results of our numerical experiments are summarized in **Table 7**.

**Table 7:** The results of numerical experiments.

Dataset	Treatment	Performance	PD-BN		HITL-BN		Delong Test	
			I-ALBI	LC	I-ALBI	LC	I-ALBI	LC
Training	Pre	AUC	0.78	0.75	0.83	0.82	0.0654	0.0875
		95% CI	0.67-0.83	0.60-0.86	0.75-0.89	0.67-0.93	NA	NA
	During	AUC	0.82	0.79	0.89	0.85	0.0253	0.0367
		95% CI	0.74-0.88	0.69-0.89	0.81-0.95	0.75-0.95	NA	NA
Testing	Pre	AUC	0.68	0.66	0.72	0.71	0.0921	0.1063
	During	AUC	0.73	0.72	0.78	0.77	0.0318	0.0406

## 4 Discussion

### 4.1 Comparison of the PD-BN and the HITL-BN approaches for class imbalance

Developed from our previous PD-BN method, the HITL-BN approach also includes feature selection and BN structure learning. To handle imbalanced data, the HITL-BN approach allows human agents to integrate the EML-based and UML-based feature selections in identifying important EKFs and non-EKFs in terms of outcome prediction. **Tables 3** and **4** show that EKFs and non-EKFs obtained from the HITL-BNs (highlighted by bold font) for I-ALBI prediction are generally ranking higher than those from the PD-BNs (emphasized by italic font) before and during SBRT respectively. Also, a similar situation can be found from **Tables 5** and **6** for LC prediction. These findings not only echo that the HITL-BNs outperform the PD-BNs for I-ALBI or LC prediction before and during SBRT as shown in **Figures 3** and **5** or **Figures 4** and **6**, but also indicate that the HITL-BN approach can increase the capability of feature selection from imbalanced data. Since the properties of imbalanced outcomes in the testing dataset are not the same as those of the training dataset, the prediction performance of the HITL-BN based outcome prediction models of the former is expectedly less than that of the latter.

The reasons for the improvement of accuracy and explainability of HITL-BN based outcome prediction models in handling the imbalanced proportion of tumors with and without LC or I-ALBI in our HCC SBRT patient dataset could be twofold. First, since traditional ML approaches for crowdsourcing labeled training examples are not effective at locating the scarce minority class examples (32), they have difficulties in handling the high-skewed domain in the real-world, and their associated outcome prediction models may have low accuracy. Active learning is designed to select representative subsets of unlabeled datasets for manual labeling, and an ML algorithm can achieve accuracy with fewer training labels if it is allowed to choose the data from which it learns (14, 33). Originating from active learning, our HITL-BN approach intends to manually label important EKFs and non-EKFs based on their strong relevance to an outcome or/and their importance in the outcome prediction, which is intended to improve the prediction of the imbalanced LC classes and I-ALBI classes. Secondly, while EKFs play an important role in the HITL-BN approach due to its explainability to gain physicians’ trust in clinical decision making, not all of them are ranked at the top of an integrated feature ranking list. They are evenly distributed into the ranking list as shown in **Tables 3**-**6**. Only the top-ranked EKFs that are strongly related to the outcome were selected to build an initial HITL-BN. In the meantime, top-ranked non-EKFs have potential to improve the accuracy of the initial HITL-BN model as well. However, given the selected EKFs in the initial model, only the non-EKFs with strong relevance to these EKFs can improve its prediction performance. Our HITL-BN approach is designed to determine the important EKFs or/and non-EKFs from integrating the EML-based and UML-based feature selection methods and maximizing the prediction performance of the developed BNs through feedback. The focused, interactive, incremental process to improve the accuracy and explainability of an outcome prediction model can be considered as an extension of cost-sensitive learning, which is one of traditional methods for class imbalance (14, 34, 35).

As some EKFs may be missing or not available in clinical practice, the HITL-BN approach can skip these EKFs or investigate the EKFs that physicians are most interested in along the integrated ranking list for the outcome prediction model development. The purpose of this study is to verify whether the HITL-BN approach can significantly improve the performance of HCC SBRT patients’ outcome prediction models or not based on imbalanced data compared to the PD-BN method. The HITL-BN approach based on two UML approaches with RF and GBM had been implemented in our numerical experiments to test the hypophysis. Our choices of these two because they resemble BN in terms of graph/tree structures. However, if the number of UML algorithms increases, whether the predictive power of the HITL-BN based outcome prediction models could be improved or not and how much it can be improved are interesting research topics that beyond our current scope and we would like to explore in the next step.

Our numerical experiments on developing the HITL-BN based outcome prediction models for HCC SBRT patients have shown that human intelligence can positively augment machine intelligence, and the assistance of human agents involved in the learning phase can enhance the capability of learning from imbalanced data. However, our study still has limitations in terms of small sample size and the assumption of two or more independent lesions in an HCC patient. In the next steps, in addition to developing more robust HITL-BN approaches by removing the within patient tumor independence assumption and conducting further external independent validations, we plan to explore an interactive human-computer interface *via* the HITL-BN approach to conduct prospective personalized SBRT trials for improving HCC patients’ radiation treatment outcomes.

### 4.2 The explainability of the HITL-BNs for HCC SBRT patient outcomes prediction

In addition to outperforming the PD-BN based outcome prediction models in terms of accuracy, the HITL-BN based outcome prediction models also have a better explainability due to the incorporation of the EKFs in their model buildings. The biophysical pathways displayed in our HITL-BNs for I-ALBI prediction before SBRT are supported by cited literatures. Since a longitudinal increase in the ALBI score is closely associated with non-malignancy-related mortality and quality of life (36), the incorporation of mid-treatment change in ALBI in addition to baseline ALBI improves the ability to predict treatment-related toxicity in patients with HCC receiving SBRT (13). Then *change* in albumin–bilirubin score (*ALBI*) score at three months after SBRT were used in many studies to capture acute toxicity occurring <90 days after SBRT (37). Studies showed that repeated SBRT in patients with advanced liver *cirrhosis* seems to exhibit higher hepatic toxicity (38), and the severity of hepatic cirrhosis is a major prognostic factor for radiation induced liver disease (39). Also, researchers found out that direct total bilirubin and total bilirubin are not related to delivery dose, and *age* is a significant predictive factor for radiation-induced liver injury based on univariate analysis of clinical factors (39). Moreover, an elevation in alkaline phosphatase (*alkphos*) of at least 5-fold and/or that of *bilirubin* of at least 3-fold compared to either the upper normal limit or the pretreatment level corresponding to grade 3 or higher hepatic toxicity without disease progression within 3 months after SBRT is one of the conditions to define radiation-induced liver disease (40).

The following findings from literatures support the biophysical pathways displayed in the HITL-BN for I-ALBI prediction during SBRT. Increasing *mean liver dose* was associated with larger increases in toxicities (41). As the percentage of retained ICG at 15 minutes, *ICGR15*’s normal value would be in the range of 4–10% (42). While baseline values of ICGR15 may be associated with the development of radiation induced liver disease, *the change of ICGR15* after radiation therapy appears to be most indication of the toxicity (43, 44). There may exist prognostic significance of baseline serum sodium value (*pre_Na*) in HCC patients complicating with liver cirrhosis, and lower serum sodium concentration is a useful predictor for these patients (45). The time course of changes of the liver function after SBRT was analyzed in patients treated for non-resectable HCC. Albumin was the only blood test that changed systematically during a three-month period, and it stabilized thereafter, which indicates the *decrease in albumin* reflects a minor radiation-induced liver disease (46). Model for end-stage liver disease (MELD) is a scoring system used to predict three-month mortality in patients with advanced liver disease (47). An increase in MELD score is associated with a decrease in residual liver function or deterioration in liver function (48).

Moreover, our HITL-BNs to predict LC before and during SBRT are endorsed by the following recorded observations. Higher *treatment dose* was associated with improved freedom from *local progression* (41) (49). Larger *GTV* volume was significantly associated with a higher risk of death (39). While increased TGF-beta signaling has demonstrated radiation resistance (50), study shows that inhibition of *TGF-beta* stops disease progression in liver metastases from colon cancer (51) (52). Incorporation of *ICGR15* variables significantly improves the prediction of post-SBRT liver function. The use of ICGR15 can facilitate the delivery of the maximum safe dose of radiation for patients with hepatocellular carcinoma and has the potential to improve uncomplicated tumor control and survival (43). Prolonged prothrombin time (*Protime*) is the most important score when determining the incidence of radiation-induced liver disease during SBRT in patients with CP-A score 6 (53). International Normalized Ratio (*INR*) is derived from Protime which is calculated as a ratio of the patient’s Protime to a control Protime standardized for the potency of the thromboplastin reagent developed by the World Health Organization. The *MELD* is used to prioritize patients for liver transplantation and includes results for creatinine, *bilirubin*, and Protime expressed as international normalized ratio (*Protime-INR*) (54). Evidence was provided that the Protime-INR was identified as the most important methodologies may influence the MELD (54). While lower MELD scores were associated with improved survival following SBRT (55), a mathematical equation based on MELD and sodium, named the *MELD-Na* score, is a feasible and independent prognostic predictor for both short- and long-term outcome predictions in patients with hepatocellular carcinoma (56). It turns out that some features related to radiation induced liver disease such as TGF-Beta, MELD-Na, Bilirubin, etc. appeared in the HITL-BN for LC prediction, and its reason may be related to the fact that liver SBRT was conducted by limiting the toxicity from therapy and not compromising the primary objective of local control.

## 5 Conclusions

In this study, we have developed a new HITL-BN approach for HCC patients’ I-ALBI or LC prediction before and during SBRT based on previous PD-BN method. In addition to incorporating EK into its feature selection process, the HITL-BN approach allows humans to participate in an outcome prediction model building process for better handling of imbalanced HCC SBRT data. Especially, we created a novel feature selection mechanism for the HITL-BN approach by integrating the prediction strength of multiple UML methods and the explainable capability of the theoretically sound MB algorithms. Numerical experiments show that the HITL-BN based outcome prediction models significantly outperform the PD-BN based models during SBRT in terms of accuracy and explainability. In addition to gaining physicians’ trust in clinical decision making, the HITL-BN approach has the potential of becoming an important component of future human-computer interface to bridge physicians and advanced ML techniques in improving HCC patients’ treatment outcomes. Our approach can be applied to the outcome prediction of treating other types of cancer, but it still needs to be validated in external further independent datasets.

## Data availability statement

The raw data supporting the conclusions of this article will be made available upon request from authors and per institutional guidelines.

## Author contributions

KC, TL, MM, RT, IE, and YL conceived of the presented idea. YL developed the theory and performed the computations. IE and DN verified the analytical methods. LD provided the testing dataset. All authors discussed the results and contributed to the final manuscript.
